# Kidney damage associated with COVID-19: from the acute to the chronic phase

**DOI:** 10.1080/0886022X.2024.2316885

**Published:** 2024-04-01

**Authors:** Yannick Mayamba Nlandu, Elliot Koranteng Tannor, Titilope Adetoun Bamikefa, Jean-Robert Rissassi Makulo

**Affiliations:** a Nephrology Unit, Kinshasa University Hospital, Kinshasa, Democratic Republic of the Congo; b Department of Medicine, Kwame Nkrumah University of Science and Technology, Kumasi, Ghana; c Directorate of Medicine, Komfo Anokye Teaching Hospital, Kumasi, Ghana; d Renal Unit, Uniosun Teaching Hospital Osogbo, Osun State University, Osogbo, Osun State, Nigeria

**Keywords:** Kidney damage, COVID-19, chronic kidney disease, acute kidney injury, SARS-CoV-2

## Abstract

Severe acute respiratory syndrome coronavirus-2 (SARS-COV-2) infection is well established as a systemic disease including kidney damage. The entry point into the renal cell remains the angiotensin-converting enzyme 2 (ACE-2) receptor and the spectrum of renal lesions is broad, with a clear predominance of structural and functional tubular lesions. The most common form of glomerular injury is collapsing glomerulopathy (CG), which is strongly associated with apolipoprotein L1(APOL-1) risk variants. These acute lesions, which are secondary to the direct or indirect effects of SARS-CoV-2, can progress to chronicity and are specific to long COVID-19 in the absence of any other cause. Residual inflammation associated with SARS-CoV-2 infection, in addition to acute kidney injury (AKI) as a transitional state with or without severe histological lesions, may be responsible for greater kidney function decline in mild-to-moderate COVID-19. This review discusses the evidence for renal histological markers of chronicity in COVID-19 patients and triggers of low-grade inflammation that may explain the decline in kidney function in the post-COVID-19 period.

## 1. Introduction

Severe acute respiratory syndrome coronavirus-2 (SARS-COV-2) infection is responsible for coronavirus disease 2019 (COVID-19), a multifaceted pathology since the description of its multisystem involvement [1]. The cellular anchor of this infection is the angiotensin-converting enzyme 2 (ACE-2) receptor which has a wide distribution throughout the human body. Its presence in renal cells, particularly tubular, glomerular and endothelial cells, justifies the various functional and histological disorders associated with COVID-19 [1]. Since the onset of the COVID-19 pandemic, a wide range of renal manifestations has been reported including proteinuria, hematuria and acute kidney injury (AKI) [2, 3]. Renal histological studies have identified acute tubular injury and collapsing glomerulopathy as the main lesions associated with COVID-19 [2,4,5]. Although COVID-19 mortality has decreased significantly in the world overtime, the challenge now is to address the long-term complications associated with COVID-19 in general and kidney damage in particular, especially as AKI is well established as a transitional phase leading to the onset or progression of chronic kidney disease (CKD) [6]. Many previous studies have summarized the characteristics of kidney injury associated with COVID-19, but those that have addressed the long-term renal consequences of SARS-Cov-2 have often been limited to reporting evidence of a high rate of decline in kidney function [7–9]. Given this poorer outcome, which is more pronounced in patients with COVID-19 than in those without, it is important to understand the pathway of long COVID in order to propose a possible renoprotective treatment and organize post-COVID-19 care. The aim of this narrative review is to provide an overview of the kidney damage associated with the acute phase of COVID-19 toward the chronic phase of the infection. We discuss the evidence for histological markers of chronicity in COVID-19 patients and induction of low-grade inflammation that may explain the decline in kidney function in the post-COVID-19 period. Therefore, we conducted a literature review for a selection of articles from the PubMed database using the following key words - kidney damage, COVID-19, acute kidney injury, chronic kidney disease and their synonyms.

## 2. Acute kidney damage associated with COVID-19

### 2.1. Acute kidney injury associated with COVID-19

#### 2.1.1. Epidemiology and characteristics of AKI associated with COVID-19

The Kidney Disease Improving Global Outcome (KDIGO) consensus on acute kidney injury (AKI) was intended to produce a reproducible and uniform definition in the face of varied definitions [10]. Since the occurrence of COVID-19, numerous studies have been conducted reporting with incidence and prevalence of AKI. Various meta-analyses on AKI associated with COVID-19 (COV-AKI) reports a frequency between 10 and 17%, with a wide range between 0.5% and 80.5% [11–14]. This discrepancy, which has also been noted in recent African and Latin American studies, is due to several factors, including the lack of agreement between the definitions used [2,15–18]. Indeed, the definition and/or detection of AKI is mainly based on acute changes in serum creatinine and therefore the frequency of creatinine tests has an impact on the detection rate of AKI. In a Chinese study, the detection rate of AKI was 0.99% according to KDIGO criteria. After adjusting for the frequency of serum creatinine testing, the incidence of AKI increased to 11.6% [4]. The characteristics of the study population in relation to the severity of the disease (the virulence of the SARS-COV-2 variant associated with the COVID-19 wave, the burden of comorbidities [age, chronic kidney disease, heart failure, diabetes mellitus, arterial hypertension, obesity], the genetic predisposition [APOL-1 polymorphism], admission to intensive care unit or not, use of mechanical ventilation with prone position or not and the income level of the country) could account for the wide variability of the frequencies of COV-AKI reported in the literature [2,8,19–21]. In a retrospective multicenter study in the USA, Chan Li et al. reported an incidence of AKI of 46% with 19% of patients requiring replacement therapy and an incidence of 76% in patients admitted to intensive care unit, with 32% of patients requiring dialysis [22]. Rosa et al. revealed the impact of the prone position used in severe acute respiratory distress syndrome (ARDS) on the occurrence of AKI in COVID-19 patients, with body mass index and low central venous pressure as determinants [23]. In fact, the prone position could be responsible for a decrease in glomerular filtration rate leading to AKI [24]. Sanchez et al. reported that AKI was more common in patients with ARDS (68% vs 53.6%, *p* < 0.001), in those requiring mechanical ventilation (91.9% vs 77.7%, *p* < 0.001), and even more so in those in the prone position (74.8% vs 61%, *p* < 0.001) [25]. Marina et al. in an observational and multinational study, reported that patients from lower-income countries were more likely to develop AKI, highlighting the impact of health system quality [20].

The incidence and severity of AKI (AKI requiring dialysis) decreased over time and across COVID-19 waves, with the first wave being more severe [2,26,27]. These results could indicate, on the one hand, a reduction in the virulence of SARS-CoV-2 due to multiple mutations and, on the other hand, the impact of vaccination in creating collective immunity beyond natural infection and the improvement in the management of COVID-19 with the introduction of corticosteroids in the treatment of severe cases of COVID-19 [28,29].

Compared to others etiologies, the incidence and severity of COV-AKI appears to be higher than that reported in other settings such as community acquired pneumonia or sepsis [30,31], especially when considering the first wave [26,27]. Kolhe et al. in a retrospective multicenter study reported an overall incidence of AKI of 16% [30]. This incidence rose to 26.2% in COVID-19 patients vs 12.4% in non-COVID-19 patients [30]. Diebold et al. in a prospective study of 507 patients associating COVID-19 with non-COVID-19 respiratory infections, also reported a higher incidence in COVID-19 patients, 30% vs 12% [31]. Requirement for dialysis was also higher in COVID-19 patients 4.4% vs 0.9% [30].

Certain characteristics of this emerging nosological entity (COV-AKI), such as the time of onset of AKI, the start of dialysis relative to the date of admission, and the percentage of kidney function recovery, may highlight the severity of the renal lesions associated with SARS-CoV-2 infection. Mohamed et al. reported a median duration of AKI onset of 1 day (IQR, 1–4) with a median time from diagnosis to dialysis initiation of 3 (IQR, 1–6) days [13]. Chan Li et al. in a cohort of 1835 patients, reported a kidney function recovery of about 65%, a low rate when compared to other causes where the frequency of recovery is around 80% with a median duration of 10 days [22].

AKI has also been reported as a strong predictor of mortality [30,32,33]. Khole et al. concluded that AKI increased the risk of mortality by a factor of 3 in a population of patients infected with SARS-COV-2 [30]. This risk was even greater for stages 2 and 3, but also when AKI associated with COVID-19 was compared with those from other causes. The usual limitations of creatinine in the early detection of AKI encourages the use of biomarkers [34]. In COVID-19, biomarkers of AKI such as functional biomarkers (cystatin c), damage biomarkers (kidney molecule injury 1: KIM-1, l-type fatty acid binding protein: L-FABP, interleukin 18: IL-18, soluble urokinase-type plasminogen activator receptor: suPAR, and neutrophil gelatinase associated lipocalin: NGAL), and stress biomarkers (tissue inhibitor of metalloproteinase 2: TIMP-2 and insuline-like grow factor binding protein 7: IGFBP7) have been shown to be effective in detecting AKI [34,35].

#### 2.1.2. Pathophysiology of AKI associated with COVID-19

Several studies have linked kidney damage to direct viral involvement of renal cells, although evidence of viral particles in kidney tissue remains controversial. Contrary to initial studies, many recent studies have failed to detect the presence of virus in kidney tissues using various specific techniques (immunohistochemistry, *in situ* hybridization and ultrastructural examination) [36,37]. The lack of orthogonal validation and rigorous controls can lead to the detection of numerous structural mimics of viral particles strengthens the hypothesis of an indirect effect of SARS-CoV-2 [4,38,39]. However, these results need to be interpreted with caution given the long delay between the onset of renal manifestations of SARS-CoV-2 and when kidney biopsy is performed, highlighting the possible rapid clearance of virus in kidney tissue [39,40]. Detection of SARS-CoV 2 could therefore be reduced over time, as was the case with SARS-CoV [39]. The median time to clearance of SARS-CoV-2 reported in the literature varies from 11 to 24 days, while the median time between symptoms and kidney biopsy was several weeks, at which time patients had a negative reverse transcriptase polymerase chain reaction (RT-PCR) [4,39,41].

The direct cytopathic effects of SARS-CoV-2 are due to the entry and replication of the virus in renal cells, altering several regulatory pathways, in particular the renin-angiotensin-aldosterone system (RAAS), with repercussions on the kallikrein-kinin system (KKS) [40,42]. The downregulation of ACE-2 by SARS-CoV-2 entry into the intracellular medium favors the accumulation of circulating angiotensin II, which has vasoconstrictive, profibrotic and pro-inflammatory effects, further reducing renal blood flow and allowing ischemia to occur [40,42]. In addition to the damage caused by the direct cytopathic effects of SARS-CoV-2 among host renal cells, the virus can also induce an exaggerated systemic immune response, known as hypercytokinemia or cytokine storm, which is responsible for haemodynamic changes, exaggerated recruitment of pro-inflammatory cells, generation of reactive oxygen species (ROS), dysregulation of the complement system and coagulopathies (mediated by the tissue factor pathway), culminating in kidney parenchymal involvement, endothelial damage and severe effects on kidney function [40,42]. The cytokine storm would be secondary to the late type I interferon (IFN-I) signaling induced by SARS-CoV 2, which promotes the accumulation of macrophages-monocytes, resulting in high levels of pro-inflammatory cytokines and chemokines or lymphopenia, a marker of immune dysregulation [40,42]. Rhabdomyolysis has also been described as an indirect effect of SARS-Cov 2 associated with AKI [40,42].

These factors specific to COVID-19 interact with the usual risk factors for AKI such as hemodynamic instability, hypoxia and sepsis, potentiated by the use of nephrotoxic drugs such as contrast agents, non-steroidal anti-inflammatory drugs (NSAIDs), aminoglycosides, vancomycin. Several risk factors have been described for COV-AKI. Kolhe et al. stated that comorbidities (advanced age, CKD, heart failure, etc.) and mechanical ventilation were risk factors for AKI [30]. Beyond the severity of the disease defined by hypoxia with or without the need for ventilator support, See Y. et al. reported drug toxicity, mainly that related to vancomycin, as a risk factor for kidney injury in COVID-19 [43].

### 2.2. Urinary abnormalities associated with COVID-19

Various urinary tract abnormalities have been reported in the course of COVID-19 and proteinuria leading the park [33]. The incidence of proteinuria varies widely between 24% and 84% with or without AKI [3, 44]. In a study of 701 COVID-19 patients, the incidence of proteinuria (semi quantitative proteinuria ≥2 +) was 8% vs. 30%, respectively, in the no AKI vs. AKI group [5]. The use of a quantitative method, proteinuria/creatinuria (P/C) ratio, in 3345 patients by Fisher et al. reported a similar trend with an incidence of 0.6% vs 3% [45]. However, proteinuria should be interpreted with caution given the acute context of COVID-19 disease. Pei et al. reported resolution of proteinuria during COVID-19 with a median duration of 12 days in 69% of patients, emphasizing the transient nature of this proteinuria [46,47].

This proteinuria is mainly of tubular origin. Korman et al. in their qualitative study of proteinuria in COVID-19 patients reported that out of 24 electrophoreses, 17 were of tubular origin (albuminuria less than 50%), and 7 cases had mixed proteinuria (albuminuria between 50 and 80%) [48]. Huart et al. specifically reported 1 α-microglobulin as the main tubular proteinuria in COVID-19(49). Proteinuria of glomerular origin has been strongly associated with collapsing glomerulopathy [50]. Proteinuria reflects dysfunction of glomerular membrane permeability or dysfunction of reabsorption by proximal tubular epithelial cells [51]. In the context of COVID-19, disruption of the glomerular capillary wall may result in COVID associated nephropathy (COVAN), whereas impairment of tubular reabsorption during acute tubular injury is also possible. Systemic inflammatory response leading to increased glomerular permeability responsible for proteinuria and preexisting chronic kidney disease are other factors implicated in the multifactorial pathogenesis of proteinuria in COVID-19(52).

Beyond its high frequency, proteinuria during COVID-19 has also been reported to be a predictor of mortality associated with or without AKI and/or microscopic hematuria [32,53]. Gross et al. reported that analysis of the admission urine sample could be used to screen for the nephritic syndrome, which, when entered into an algorithm, predicted the risk of respiratory decompensation and even mortality [53].

### 2.3. COVID-19-associated tubulopathy

The high expression of ACE2 receptors in the proximal convoluted tubule makes it an early target of SARS-COV2 infection [54]. Emerging evidences from recent studies corroborated earlier postulations by pioneer researches on COVID-19 on the existence of tubulopathy in COVID-19 mediated renal damage. Morelle J et al. had tested the hypothesis of a proximal tubulopathy among 49 patients with COVID-19(55). Their analysis reported the presence of tubular proteinuria (median P/C ratio, 0.7 g/g) in 80% of patients, associated with tubular leakage of uric acid (46%) and phosphorus (19%), without urinary glucose loss [55]. Some patients (46%) also had a urinary leakage of neutral amino acids. Moreover, these authors reported a fraction of uric acid excretion >10% in the presence of hypouricemia [36]. Kormann et al. reported the first study demonstrating acute incomplete Fanconi syndrome in patients admitted with mild, moderate, or severe COVID [56]. In their cohort of 42 patients, 75% had at least two of four defining elements of renal Fanconi syndrome (renal phosphate leakage, normoglycemic glycosuria, hyperuricosuria and proteinuria). The main abnormalities were proteinuria (88%), renal phosphorus leakage (55%), hyperuricosuria (43%) and normoglycemic glycosuria (30%) [56]. In their series, patients admitted to intensive care experienced the more severe forms of Fanconi syndrome. This tubulopathy preceded the occurrence of AKI and disappeared during the renal recovery phase. Werion et al. also reported that this tubular dysfunction was associated with the severity of COVID-19 disease with an increased risk of respiratory failure requiring mechanical ventilation [36].

These biological abnormalities (renal phosphate leakage, normoglycemic glycosuria, hyperuricosuria and proteinuria) are associated with structural lesions of the proximal tubule on postmortem examination of kidneys from patients who died of COVID-19 [28]. Indeed, histological evaluation and interpretation showed consistent evidence of tubular damage, with necrosis and loss of the brush border of the proximal tubule, as well as a significant reduction (50%) in megalin receptor expression at this level [36,37]. The electron microscopic demonstration of unusual particles as evidence of direct infection [56,57], however, has been questioned because normal structures could be mistaken for virions [58].

SARS-CoV-2 would therefore directly infect proximal tubular cells *via* the ACE2 receptor, causing proximal tubular dysfunction. The toxicity of drugs used in COVID-19 should also be added. Wongboonsin et al. have reported an interesting case of osmotic tubulopathy in the context of remdesivir use [59].

Angiotensin-converting enzyme 2 (ACE2), the ligand for viral cell invasion is expressed on both proximal and distal tubular cells [54]. This explains distal involvement in the context of COVID-19. Wan et al. reported a case of nephrogenic diabetes insipidus in the context of COVID-19 [60]. Renal tubular acidosis with hyperkalemia has often been reported in the context of heparin use [61,62].

## 3. Long-term renal complications of COVID-19

The burden associated with SARS-CoV-2 infection has been enormously felt by healthcare systems around the world [43]. While mortality has gradually declined as the virus strain has mutated, making it less virulent, the long-term effects on various organs are beginning to be felt. As far as the kidneys are concerned (with a wide distribution of ACE2), residual inflammation is a major concern, whether or not in a post-AKI context [44], not forgetting the renal histological status after severe SARS-Cov 2 infection.

### 3.1. Kidney function decline in post-COVID-19

The evolution of kidney function after AKI depends not only on the severity of the damage, but also on the nephron reserve prior to the acute episode. The kidney outcome under these conditions can be summarized as either full recovery, partial recovery defining acute kidney disease (AKD), or direct entry into chronic renal disease (CKD) [45]. This evolutionary description should also apply to COVID-19, and could even reveal a precarious kidney outcome at short term in view of its severity, well-defined by a high rate of admission to ICU and the long stay occasioned by the existence of multiple complications, including AKI. In a retrospective study, Strohbehn et al. reported a higher proportion of severe AKI (7% vs 1%) and non-recovery of kidney function at discharge (70% vs 56%) in COVID-19 patients than in those admitted for Haemophilus influenza [46,61]. The low rate of short-term recovery of kidney function in COVID-19 patients has also been reported in African populations [2]. What’s more, the population most affected by COVID-19 is likely to have reduced nephron reserves (elderly patients, comorbid patients [hypertension, diabetes mellitus, etc.]).

Huang et al. were the first to report a decline in kidney function over a long-term period of 6 months [49]. These authors reported that 35% of patients discharged following COVID-19 had a progressive decline in kidney function at 6 months, defined as a glomerular filtration rate (GFR) of less than 90 mL/min [49]. These results were confirmed over a longer period. Li et al. reported that 28.5% of patients hospitalized for COVID-19 had a GFR of less than 90 mL/min at 1 year after discharge, a significantly different frequency when comparing non-severe and severe patients [50]. Interpretation of these results should be made with caution, as on the one hand, the chosen cutoff of 90 mL/min would not reflect the reality of CKD, especially in the absence of evaluation of markers of chronicity such as proteinuria [51], and on the other hand, the existence of a subclinical state of CKD is always difficult to assess [44]. The rate of decline in kidney function in COVID-19 depends on the basis of COVID-19 severity associated or not with AKI, and compared with other causes of AKI, this rate could be more pronounced in COVID-AKI [52,53]. Bowe et al. reported that the estimated GFR decline rate varied from −3.26 in non-hospitalized patients, −5.2 in hospitalized patients to −7.69 mL/min/1.73m^2^ per year in intensive care unit (ICU) patients [63]. Regarding AKI status, Nugent et al. reported that patients with COV-AKI had a greater decline in eGFR after adjustment for baseline comorbidities (−12. 4 mL/min/1.73 m2/year) and that this decline was faster in patients with COV-AKI compared to patients with non-CoV-AKI [64]. Advanced age, severe AKI, hypertension and male gender have also been reported as risk factors for progression to CKD after an episode of COV-AKI [55].

**Figure 1.**
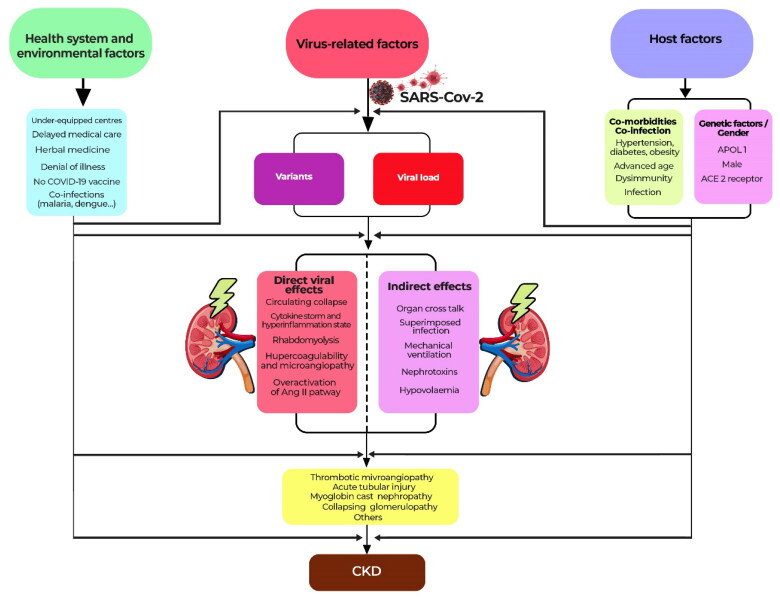
Kidney damage associated with COVID-19: from the acute to the chronic phase.

Apart from AKI, the post-COVID-19 state may involve a risk of decline in kidney function. On the one hand, the description of the de-novo development of certain comorbidities such as hypertension, diabetes mellitus and dyslipidemia in post-COVID-19 could not only participate in the development of CKD, but also already be a subclinical manifestation of CKD [47]. On the other hand, Yende et al. described that several direct or indirect effects of SARS-Cov-2 infection could persist during the post-discharge recovery phase, leading to a recurrence of septic and AKI episodes and thus an increased risk of CKD [44]. Long et al. summarized the mechanisms leading to CKD post COVID-19 as unresolved tubular lesions, the existence of micro and/or macro vascular lesions and podocytopathies, with collapsing glomerulopathy at the top of the list [48]. Figure 1 summarizes the factors that explain the kidney damage induced by SARS-CoV-2.

### 3.2. Renal histological markers of chronicity in COVID-19 patients

COVID-19 kidney biopsy findings associated or not with AKI severity may explain the decline in kidney function after COVID-19 infection. Acute tubular injury (ATI) and collapsing glomerulopathy (CG) are the two main histological lesions reported in the literature [4]. The clinical phenotype associated with CG is more severe (AKI KDIGO 3, requiring renal replacement therapy [RRT]) and may predict a poor renal outcome. Volbeda et al. reported a prevalence of 93% AKI KDIGO 3 and 72% RRT in covid-19 patients with GC [65].

Renal histological markers of chronicity in COVID-19 patients have also been reported, including interstitial fibrosis and tubular atrophy (IFTA), whose severity correlates better with renal progression. In a systematic review of 39 different studies, Volbeda et al. reported that IFTA are features strongly associated with a decline in GFR over time [65]. These authors reported a high IFTA proportion of 72% in a series of 89 kidney biopsies and the severity of IFTA correlates with renal recovery (recovery in 5/7 with <30% IFTA vs 0/4 with >30%) [65]. Similar results were reported in an American series with lack of kidney recovery in COVID-19 patients with more than 30% IFTA [66]. Interstitial fibrosis and tubular atrophy (IFTA) is the most critical feature of CKD and is superior to glomerular pathology in predicting decline in glomerular filtration rate (eGFR) [67]. IFTA is the result of an orchestration of a variety of cell types and molecular pathways. Tubular epithelial cells, fibroblasts, fibrocytes, myofibroblasts, monocytes/macrophages and mast cells are involved. The cellular and molecular interactions are complex and incompletely understood. A large number of molecular mediators are involved through pathways such as transforming growth factor beta (TGF-β), bone morphogenic protein (BMP), platelet-derived growth factor (PDGF) and hepatocyte growth factor (HGF) [68]. Jansen et al. reported in an experimental study on organoids kidney a profibrotic response to the SARS-CoV-2 infection [69]. This study may support the hypothesis that, in the absence of AKI, which is known to be a transition state to CKD, SARS-CoV-2 infection itself may put patients at risk of kidney function decline. However, Schmidt-laubert et al. did not report this progressive decline over 9 months of follow-up in non-severe patients, even using Dickkopf-related protein 3 (DKK3), a profibrotic marker associated with decline in renal function [70].

Collapsing glomerulopathy (the most severe form of focal segmental glomerulosclerosis), which presents as an acute glomerular lesion, is also associated with a poor renal outcome, with a renal survival time of 13 months [71]. Most cases present with refractory proteinuria, severe loss of kidney function, and progression to RRT dependence [71]. In the context of COVID-19, this poor outcome is also reported in the recent literature. Kudose et al. reported a series of 23 patients with COVID associated nephropathy (COVAN) with CKD at follow-up, although half of the patients who initially required dialysis achieved dialysis independence [72]. Giannini et al. in a larger series of 43 patients, reported the same result, progressive CKD in all patients included in the series with a mean eGFR of 26.6 ± 14.2 mL/min and a mean serum creatinine of 3.1 ± 1.9 mg/dl [73]. The poor prognostic indicators in this series were glomerulosclerosis (*p* = 0.0005), moderate to severe interstitial fibrosis and tubular atrophy (*p* = 0.034) and severe arteriolar hyalinosis (*p* = 0.039) [73].

Preexisting chronic lesions are of concern because they contribute to the decline in kidney function at a rate commensurate with the initial chronic lesion. Volbeda et al. reported in their series a high frequency of chronic histological lesions (83%) with a prevalence of 63% for glomerulosclerosis and 63% for atherosclerosis [65].

### 3.3. Residual low-grade inflammation in COVID-19 patients

Low-grade and chronic systemic inflammation is a condition characterized by persistent low to moderate levels of circulating inflammatory markers [74]. The role of this inflammation in the pathogenesis and progression of CKD has been reported in many studies [75]. Following SARS-CoV-2 infection, which is an acute disease, there may be a shift in the inflammatory response from short-term to long-term (chronic inflammation). The link between these two inflammatory states is provided by damage-associated molecular patterns (DAMPS) released by damaged cells or tissues which are responsible for immune dysregulation leading to uncontrolled inflammation and multi-system organ damage [76]. The formation of DAMPS during COVID-19 and the long-term persistence of SARS-CoV-2 viral remnants reported in numerous sites, including the kidneys, are the main triggers of an aberrant immune response responsible for low grade inflammation (LGI), which can persist over time [76,77]. The evidence of persistent LGI was reported in the context of SARS-CoV-2 infection [77–79]. Doykov et al. analyzing 96 proteins associated with the immune response, observed a significant residual inflammatory response even 40–60 days after COVID-19 infection [79]. These authors showed an increase in a group of biomarkers such as the cytosolic protein N-Myc downstream regulated gene 1 (NDRG1) and the mitochondrial protein peroxiredoxin 3 (PRDX3), which are involved in inflammation and the stress response respectively [79]. Using a targeted single-cell multiomics approach, Zhang et al. showed that SARS-CoV-2 infection leads to a prolonged change in the gene expression profile of circulating T, B and NK cells and monocytes, indicating a pro-inflammatory state [77]. The altered transcriptional profile, which was not observed in response to influenza infection or sepsis, was detected in COVID-19 patients for at least 2 months after the onset of disease symptoms [77].

Persistent low-grade inflammation has been recognized as an important component of the CKD scenario, leading to fibrosis and loss of kidney function [74]. It plays a crucial role in the pathophysiology and progression of the disease and has a major impact on its complications [74]. Stuveling et al. showed that in a large non-diabetic population, low-grade inflammation, as measured by a highly sensitive CRP assay, was independently associated with reduced kidney filtration [80]. Inflammation is reported to contribute to glomerular injury through the infiltration of inflammatory cells such as monocytes and macrophages, which stimulate mesangial cell proliferation, leading to kidney scarring [81]. A low-grade inflammation may therefore be a possible explanation for the decline in kidney function following infection with the SARS-CoV-2 virus. However, to our knowledge, few studies have attempted to investigate this possibility, limiting themselves to analyzing the association between decline in renal function and inflammatory markers on admission, such as CRP [82].

## 4. Conclusion and perspectives

SARS-CoV-2 infection is not exclusively a respiratory pathology. To date, functional and structural kidney damage is well established. ACE-2 receptor, as anchor point of the virus, is widely distributed at the level of proximal tubular cells and somewhat podocytes explaining that the proximal tubular damage is the most frequent. SARS CoV-2 infection is thought to be responsible for a residual inflammatory state and fibrotic renal lesions those are the breeding ground for chronic kidney disease especially after an episode of AKI. In this context, COVID-19 patients should be classified as a population at risk of kidney disease although studies of kidney sequelae in patients with non-severe COVID-19 remain conflicting to date [56,57].

While early detection of CKD is needed to improve outcomes, renoprotective medications remain a challenge. Bowe et al. reported in a large international multi-centre study that remdesivir was associated with better recovery of kidney function but had no protective effect on kidney function decline over time, suggesting the existence of another mechanism that could explain kidney damage in patients with long-term COVID-19(63). An abnormal immune response or autoimmunity and persistent inflammation have already been highlighted, and the DAMPS pathway as a potential target may serve as a basis for future research on long COVID, especially as there are currently no effective drugs for renal fibrosis [58,83].

With a large number of patients infected with COVID-19 throughout the world, there is a reel need to organize a post COVID-19 kidney surveillance and one way to attend this objective is to create a registry of kidney disease in patients who suffered from COVID-19 with follow-up over time based on assessment of kidney function and urine analysis including proteinuria.
